# Adherence to Various Dietary Quality Indices in the New England Centenarian Study

**DOI:** 10.1093/geroni/igaf122.2015

**Published:** 2025-12-31

**Authors:** Erfei Zhao, Emma Schluter, Naglaa El-Abbadi, Kyla Shea, Stacy Andersen, Thomas Perls, Paola Sebastiani, Andres Ardisson Korat

**Affiliations:** Human Nutrition Research Center on Aging, Tufts University, Boston, Massachusetts, United States; Harvard University, Boston, Massachusetts, United States; Jean Mayer USDA Human Nutrition Research Center on Aging, Boston, Massachusetts, United States; Tufts University, Boston, Massachusetts, United States; Boston University, Boston, Massachusetts, United States; Boston University, Boston, Massachusetts, United States; Tufts Medical Center, Boston, Massachusetts, United States; Tufts University, Boston, Massachusetts, United States

## Abstract

Centenarian offspring (CO) in the New England Centenarian Study (NECS) typically show survival and health advantages, yet little is known about their dietary intake patterns. In this study, we characterized participants’ diets according to four established patterns: Alternative Healthy Eating Index (AHEI), Healthy Eating Index (HEI), Mediterranean-DASH (Dietary Approaches to Stop Hypertension) Intervention for Neurodegenerative Delay (MIND), and Planetary Health Diet Index (PHDI) in CO and a referent group and compared them to national dietary surveys. NECS participants (335 CO; 128 controls; mean age: 73.6 years; 55.1% women) completed the Harvard 131-item food frequency questionnaire in 2005. We assessed each index according to published scoring systems and performed linear regression to examine differences by CO status, sex, age, and education, and compared mean scores to nationally representative data (e.g., National Health and Nutrition Examination Survey [NHANES ]) for older individuals. We found no significant differences between CO and referents across all four indices. However, women had slightly higher AHEI and HEI scores than men (P < 0.05). Younger participants scored higher on AHEI, while education was positively associated with all indices (P < 0.01). Compared to NHANES participants of similar ages, NECS participants scored modestly higher on all indices; however, intakes of whole grains and dairy were below national averages, while seafood protein and added sugar were higher. In summary, CO showed no advantage in dietary quality compared to controls, whereas sociodemographic factors were significant predictors of eating patterns. Future research should explore the diet-physiology-lifestyle interaction and its combined effect on CO’s survival advantage.

